# 

**Published:** 1898-04

**Authors:** 


### CONTENTS.

**ORIGINAL COMMUNICATIONS—**

The Pathology and Diagnostic Value of Pain. By J. Clarence Johnson, M.D., Atlanta, Ga .......... 73
Some Cases of Drug Habit. By C. C. Stockard, M.D., Atlanta, Ga .......... 80
Some of the Early Experiences of an Old Physician. By G. G. Roy, M.D., Atlanta, Ga .......... 86
Some Cases of Drug Eruptions. By W. H. Hudson, M.D., LaFayette, Ala .......... 90
Remarks on Gude's Pepto-Mangan. By T. V. Hubbard, M.D., Atlanta, Ga .......... 93

**SOCIETY REPORTS—**

Atlanta Society of Medicine .......... 96

**CORRESPONDENCE—**

New York Letter .......... 105
Hypodermic Needles .......... 110

**EDITORIAL—**

The Medical Association of Georgia .......... 111
Meeting of the Southern Section of the American Laryngological, Rhinological and Otological Society .......... 114
Commencements .......... 115

**MEDICAL ITEMS** .......... 118
**BOOK REVIEWS** .......... 122
**SELECTIONS AND ABSTRACTS** .......... 126
**MEDICAL ITEMS**—Continued .......... x, xi, xii, xiii, xiv, xvi, xvii
**ADVERTISERS' INDEX TO ADVERTISEMENTS** .......... xviii
**CLASSIFIED INDEX TO ADVERTISEMENTS** .......... xix

